# Retinal Sensitivity Loss Correlates with Deep Capillary Plexus Impairment in Diabetic Macular Ischemia

**DOI:** 10.1155/2019/7589841

**Published:** 2019-10-13

**Authors:** Fabio Scarinci, Monica Varano, Mariacristina Parravano

**Affiliations:** IRCCS—Fondazione Bietti, Rome, Italy

## Abstract

**Purpose:**

To assess retinal sensitivity and retinal morphologic changes of capillary nonperfused areas in diabetic macular ischemia.

**Methods:**

Observational cross-sectional study. Patients were examined at IRCCS—Bietti Foundation, Rome, Italy. Fourteen consecutive diabetic eyes showing outer retinal changes on spectral domain optical coherence tomography B-scan were included. Ten eyes of ten diabetic patients with normal outer retinal structure on SD-OCT were included as controls. All eyes underwent optical coherence tomography angiography (OCTA) and MP1 microperimetry. To explore the outer retina findings and localized areas of capillary nonperfusion at the superficial and deep capillary plexus, we used the Spectralis HRA + OCTA (Heidelberg Engineering, Heidelberg, Germany). The B-scans as either normal or having outer retinal disruption and the enface images at the level of the superficial and/or deep capillary plexus were evaluated to identify areas of capillary nonperfusion.

**Results:**

Fourteen eyes of 12 consecutive type 2 diabetic patients with outer retinal disruption on SD-OCT showed that areas of capillary nonperfusion of the deep capillary plexus were colocalized to areas of reduced retinal sensitivity.

**Conclusions:**

On optical coherence tomography angiography, areas of capillary nonperfusion of deep capillary plexus due to macular ischemia are associated with photoreceptor structural abnormalities and retinal sensitivity loss on microperimetry. This highlights that the health status of deep capillary plexus and not only the choroid is important to the oxygen requirements of the photoreceptors in patients with diabetic macular ischemia. Also, the anatomical and functional consequences of these findings might help to explore the efficacy of new therapy into the macular area in clinical practice.

## 1. Introduction

Diabetic macular ischemia (DMI) and foveal avascular zone (FAZ) enlargement are two important clinical findings and biomarkers of poor visual function in patients with diabetic retinopathy (DR).

While the initial change of the normal FAZ size and contour might be not often correlated with clinically significant reduction in visual acuity, the decreased perifoveal capillary blood flow and oxygen delivery to the fovea lead to visual complain in working-age individuals with time [1–3].

Optical coherence tomography angiography (OCTA) is a new device that provides the ophthalmologists with the capability to explore both the superficial capillary plexus (SCP) and deep capillary plexus (DCP) of the retina. Recently, several studies [4, 5] using either spectral domain optical coherence tomography (SD-OCT) or OCTA reported on the relationship between vascular abnormalities into the foveal and perifoveal region and the outer retinal structure in diabetic retinopathy at the level of both SCP and DCP [6]. Changes at the level of the DCP were observed even before the presence of any characteristic sign of diabetic retinal disease [5]. Furthermore, more advanced retinal microvascular impairment into the macula has been correlated with the outer nuclear layer as well as inner/outer segment (IS/OS) junction damage in diabetic retinopathy [6].

Specifically, changes at the level of the DCP on OCTA seem to be associated with photoreceptors loss [7]. However, since there is not yet a consensus whether these retinal findings should be considered only anatomical retinal changes or associated with a functional impairment, multimodal imaging studies are needed to assess any potential pathological correlation between the DCP impairment and functional alteration into the macular area in patients with diabetic macular ischemia.

This far, the most common methods used to explore the visual function are best-corrected visual acuity measurements (BCVA) and microperimetry. The microperimetry technology offers repeatable functional testing of precise retinal points in the macular area through eye movement tracking and can be used in assessing baseline visual function by means of retinal sensitivity [8–10].

In a previous study, areas of capillary nonperfusion resulting from severe nonproliferative and proliferative diabetic retinopathy evaluated by means of fluorescein angiography showed morphologic changes of the vascular retinal structure correlated with a loss of retinal sensitivity [11].

The aim of this study is to explore the relationship between structural and functional retinal impairment at the level of the SCP and DCP, evaluated by means of OCTA and microperimetry, in diabetic patients with outer retinal changes revealed using SD-OCT.

## 2. Patients and Methods

### 2.1. Subjects

This observational cross-sectional case-control clinical study was notified to the local ERB (Ethical Review Board) and performed in agreement with the Declaration of Helsinki for research involving human subjects and in accordance with the Italian law on Privacy and Data Protection. Informed consent was obtained from all study participants.

The study population included fourteen consecutive eyes of 12 patients diagnosed with different stages of diabetic retinopathy, ranging from minimal nonproliferative DR to high risk and quiescent proliferative DR.

The diagnosis of DR was based on a comprehensive medical and ophthalmic history and full ophthalmologic examination including best-corrected visual acuity (BCVA), external slit-lamp, and fundus examination. Two experienced examiners (MP and FS), based on the analysis of color fundus photographs, classified the eye as no DR (no abnormalities), nonproliferative DR (NPDR), or proliferative DR according to the modified Early Treatment Diabetic Retinopathy Study (ETDRS) retinopathy severity scale and analyzed the B-scan OCT images to explore the presence of edema [3, 12].

**Figure 1:**
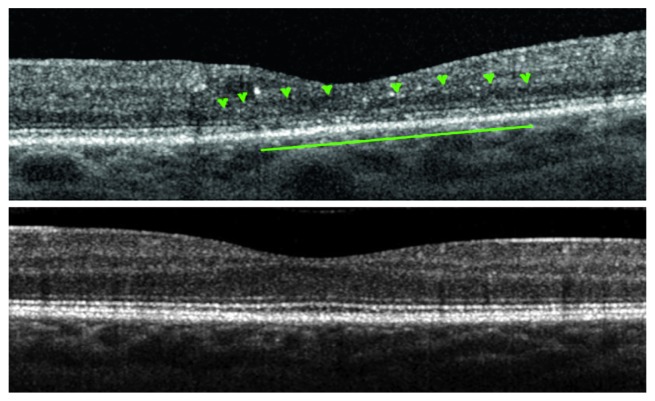
Spectral domain optical coherence tomography macular B-scans showing the comparison between a diabetic eye (above) with outer nuclear layer thinning (green arrowheads), external limiting membrane and inner and outer segment junction abnormalities (green line), and a control with normal outer retina appearance (below).

Enrollment criteria included a diagnosis of outer retinal changes/photoreceptor disruption, including at least one of the following abnormalities: outer nuclear layer (ONL) thinning, external limiting membrane (ELM), or IS/OS junction abnormalities revealed by means of SD-OCT macular B-scan, as defined in previous similar studies (Figure 1 as an example) [6, 7, 13].

Scans, where the presence of focal macular edema or microaneurysm/exudates was noted, were not considered for the analysis. Ten eyes of ten age-matched type 2 diabetic patients with proliferative and nonproliferative diabetic retinopathy with the normal outer retinal structure on SD-OCT were included as controls.

Exclusion criteria included eyes that had received intravitreal antivascular endothelial growth factor or previous history of metamorphopsia. Patients with previous surgical retinal repair and history of any other retinal disease or laser treatment at the posterior pole were not considered.

Significant cataracts or OCTA images and part of the images that had movement or shadow artefacts in the area of interest were also excluded.

Patients underwent a full ophthalmological examination, including best-corrected visual acuity (BCVA), slit-lamp examination, and ophthalmoscopy. Based on the analysis of color fundus photographs, the diabetic retinopathy stage of the eye included in the study was classified according to the modified Early Treatment Diabetic Retinopathy Study retinopathy severity scale [12].

### 2.2. Image Collection and Analysis

SD-OCT and OCTA images were obtained using the Spectralis HRA + OCTA (Heidelberg Engineering, Heidelberg, Germany). Spectralis has an A-scan rate of 70,000 scans/s, using a light source centered on 870 nm, with an axial and transverse resolution of 3.9 and 6 *μ*m in tissue.

The images were generated using the horizontal SD-OCT cross-section (13 lines spaced 250 *μ*m apart). To get the maximum quality, 25–30 frames were averaged for each B-scan.

The SD-OCT scans for each patient were qualitatively evaluated as normal or as having thinning of the inner retina and/or outer retina. Outer retinal changes were defined as focal thinning of the outer nuclear layer (ONL), disruption of the inner segment-outer segment (IS/OS) junction, or thinning of the OS-retinal pigment epithelium junction.

An OCTA scan pattern of 10 × 5 degrees (44 × 1.5 mm; consisting of 131 B-scans separated by 11 *μ*m) centered on the fovea was acquired. The OCTA image automated real-time mode was settled at 35 (frames averaged per B-scan). The SCP and the DCP enface images were visualized automatically segmenting 2 separate slabs defined by the arbitrary segmentation lines created by the software of the device: a superficial slab extending from the inner limiting membrane (ILM) to the outer border of the inner plexiform layer (IPL) for the SCP and a deeper one from the outer border of the IPL to the outer border of the outer plexiform layer (OPL) for the DCP.

All images were reviewed to confirm consistent segmentation by the automated instrument software. In all images, automated segmentation was correct. Poor-quality images (quality index lower than 35 dB) were excluded. The projection-resolved optical coherence tomographic angiography tool and the enface images of the SCP and DCP automatically generated by the inbuilt software of the device were used for the analysis of the images.

The OCTA images were examined by the agreement of two independent masked graders (FS and MP) to define the presence and location of capillary nonperfusion areas and/or reduced capillary density and/or larger perivascular space in either the SCP or DCP. In addition, also a qualitative evaluation of the FAZ at the level of SCP and DCP, which was defined as normal or enlarged, was performed. Incidents of disagreement between graders were resolved by an open discussion to reach consensus.

## 3. Microperimetry Testing

Microperimetry testing was performed with the MP1 microperimeter device (Nidek Technologies, Padova, Italy). Microperimetry testing parameters were a grid of 41 stimuli covering the central 10° (centered on the fovea), stimulus size Goldmann III with 200 ms projection time, white monochromatic background at 4 apostilb, and a bright red cross of 2° in size was used as the fixation target. A 4-2 strategy was used with an automatic eye tracker that compensates for eye movements. The fellow eye was patched. Pretest training was performed, and a 5-minute mesopic visual adaptation was allowed before starting the test. All subjects underwent microperimetry with dilated pupil. If no threshold value was detected, the corresponding area was defined as absolute scotoma. Two examinations were performed for each patient, and the second one was considered for the analysis.

Both examinations, OCTA and microperimetry, were performed during the same day.

The following parameters were quantified: mean retinal sensitivity (RS), foveal RS (consistent with the stimulus located in the center of the grid), and mean RS of central 1° (containing the remaining stimuli located in the central 1° area) [14]. A 18 dB value is considered as normal retinal sensitivity one [15].

To perform point-by-point correlation between the capillary nonperfusion on OCTA and retinal sensitivity on microperimetry, we superimposed the vascular landmarks of the near infrared images of the OCTA onto the vascular landmarks of the color image on microperimetry. The in-built software of MP1 microperimetry that allow 3-point registration was used. Then, the SCP and DCP of the OCTA were analyzed, thus allowing point-by-point correlations between the two microvascular plexus and areas of retinal sensitivity tested.

Two independent masked graders (FS and MP) masked to any associated information analyzed Spectralis HRA +OCT and OCTA images.

## 4. Results

Fourteen eyes of 12 consecutive type 2 diabetic patients were enrolled. The ages of the patients ranged from 48 to 69 years (ten male and two female) with a mean diabetes duration (±SD) of 10.3 years (±5.2) and a mean HbA1c (±SD) equal to 9.1% (±1.2).

Of the fourteen eyes, seven eyes had proliferative diabetic retinopathy and seven eyes had severe nonproliferative DR.

Snellen best-corrected visual acuity of the eyes included in this study varied from 20/20 to 20/40, and foveal thickness ranged from 230 to 342 *μ*m (mean ± SD 275 ± 29.2 *μ*m).

Mean RS fluctuated between 9.2 and 15.8 dB (mean ± SD 12.76 ± 2.35 dB) and foveal RS between 5 and 13 dB (mean ± SD 11.07 ± 2.56 dB), while retinal sensitivity of 1° centralis ranged from 3.10 to 15.9 dB (mean ± SD 11.68 ± 3.78 dB). Case 1 with PDR showed the thinnest CRT along with the lowest values of MRS, foveal RS, and retinal sensitivity of 1° centralis. This latter showed also the presence of three dense scotomas (0 dB) in the scan was included in the analysis (see Tables 1 and 2 for details).

### 4.1. Image Analysis

All fourteen eyes in this study showed on the SD-OCT B-scan considered in the analysis a spectrum of ONL thinning and outer retinal changes/photoreceptor disruption, including ELM and IS/OS junction abnormalities. Four eyes showed an irregular retinal contour at the level of the innermost retinal layers.

**Figure 2:**
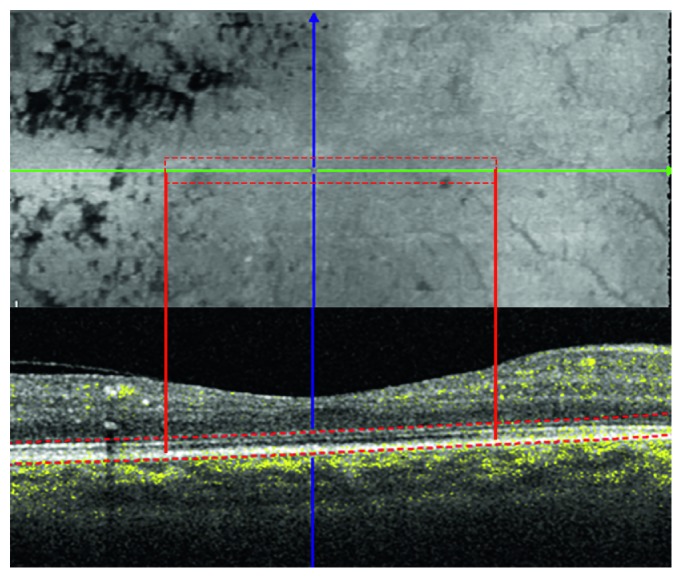
B-scans with inner and outer segment junction alteration (red box and red lines) passing below and over the exudates. The corresponding enface image shows that there is not a shadow effect of the exudates, also visible as dark spots, at the level of the photoreceptor.

Case 9 presented large hard exudates in the temporal perifoveal area. However, shadow artifacts did not determine the changes of the outer retina found in the SD-OCT B-scan included in the analysis, as shown in Figure 2.

All fourteen eyes showed capillary derangements on OCTA including an irregular and enlarged contour of FAZ along with reduced capillary density, appearing as areas of capillary nonperfusion of either SCP or DCP or both (see Table 2 for details).

Point-by-point correlation between areas of capillary nonperfusion on OCTA and retinal sensitivity map showed an exact correspondence between areas of capillary nonperfusion and areas of reduced sensitivity, as shown by the MP1 mapping image.

**Figure 3:**
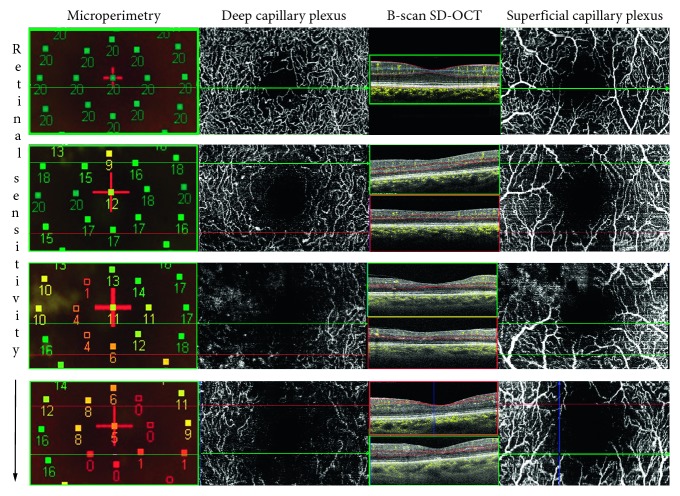
Microperimetry map of the 3 × 3 area spanned with optical coherence tomography angiography (OCTA) in diabetic patients with various stages of superficial and deep capillary plexus impairment and retinal sensitivity reduction. The first case on the top of the image represents the normal control with no retinal sensitivity and no retinal vascular and anatomical abnormalities. Left column: microperimetry maps show a progressive reduction of the retinal sensitivity from the top to the bottom. Middle column: enface deep capillary plexus images highlighting the abnormalities of no flow areas at the level of the DCP corresponding to the points of reduced retinal sensitivity. The red and green lines indicate the lower reflectivity of the inner segment-outer segment and outer segment-retinal pigment epithelium junctions corresponding to zones of reduced capillary flow signal on the structural B-scan along with alteration of the normal retinal contour. Right column: enface superficial capillary plexus images showing the overlying area in cases with or without involvement of the vascular damage.

Ten eyes of ten age-matched patients with nonproliferative and proliferative diabetic retinopathy without any outer retinal abnormalities on SD-OCT had normal macular DCP on OCTA (with or without capillary nonperfusion in the SCP) along with normal retinal sensitivity tested on microperimetry. These eyes served as controls. There was no disagreement between the readers on the analysis of images (Figure 3).

## 5. Discussion

The use of multimodal imaging in this series highlights that there is a close spatial correspondence between outer retinal disruption and DCP impairment along with retinal sensitivity reduction in patients with diabetic macular ischemia, suggesting a possible causal relationship.

In previous studies, we have already demonstrated that photoreceptor disruption on SD-OCT in eyes with diabetic macular ischemia corresponds to areas of capillary no flow at the level of DCP on OCTA [6]. However, there is not a clear consensus whether this could represent only anatomical retinal changes or also correspond to a real functional impairment.

Interestingly, Unoki et al. have reported correspondence between areas with reduced retinal capillary perfusion of the superficial capillary plexus on fluorescein angiography and regional visual field sensitivity abnormalities in diabetic retinopathy [11]. Specifically, they found that retinal sensitivity explored with MP1 was markedly reduced in the area of capillary nonperfusion secondary to advanced stages of diabetic retinopathy associated with disorganization of the retinal structure on OCT [11].

Nonetheless, in this cohort of patients, the authors could not explain since retinal sensitivity adjacent to areas of capillary nonperfusion was already reduced [11]. Our findings confirmed that deep capillary ischemia in the macular area in patients with the advanced stage of diabetic retinopathy lead to the disruption of the outer retina in diabetic patients with macular nonperfusion. The presence of reduced retinal sensitivity value seemed also being correlated with DCP impairment. However, looking at the overall data, in spite of diffuse DCP damage, only few dense scotoma (0 dB) was found, meaning that the choroid support to the function of the photoreceptors was still of value in this cohort of patients. (Figure 3)

By confining our analysis to SD-OCT B-scan of eyes without diabetic macular edema, we excluded the potential confounding effects of diabetic macular edema.

Only case 9, as described in the results, had large hard exudate in the temporal perifoveal area.

We cannot totally exclude that in this case, even if the B-scan selected for the analysis passed below and above the exudates, they might have partially influenced the decreasing of the retinal sensitivity in the adjacent areas.

Interestingly, case 1 with PDR showed the thinnest CRT and the highest value of HbA1c associated with the lowest values of MRS, foveal RS, and retinal sensitivity of 1° centralis. In this latter, we observed the presence of three dense scotoma (0 dB) points and an abnormal foveal contour.

In our cohort, all patients included had relatively good visual acuity values ranging from 20/40 to 20/20. However, as discussed in previous papers, BCVA is solely the expression of foveal function and may underrepresent the whole macular function and visual complain of the patients, whereas macular sensitivity, evaluated by means of microperimetry, can better quantify macular dysfunction in patients with relatively preserved visual acuity [16].

As 10° of visual angle equates to approximately 2.88 *μ*m on the human retina, microperimetry in this area best represents retinal sensitivity in the foveal and perifoveal areas most directly affected by diabetic retinopathy.

More recently, using OCTA, Dupas et al. found that, in patients with type 1 diabetes without macular edema and bilateral severe nonproliferative or proliferative diabetic retinopathy, functional abnormalities, expressed as a decreased VA, were associated with the degree of capillary loss in the deep capillary complex [17]. Furthermore, other studies using adaptive optics [7, 18, 19] revealed that the presence of pathologic changes in the cone mosaic along with the extent of photoreceptor loss was positively correlated with DR severity and DCP drop out.

Based on these findings, it is interesting to speculate that it could be a critical hypoxic threshold for the photoreceptor causing visual loss. Although in the normal retina the DCP contributes only for 15% of the outer retina supply [20], in particularly of the metabolic demand of the inner segments, in pathological conditions, DCP impairment seems to play detrimental effects on the middle retina as well as on the functionality of the photoreceptors [6].

Our findings do not clarify whether neurodegenerative events could precede the microvascular changes. With regard to this, looking at the B-scan images of patients included, only four eyes showed alteration of the inner retinal contour. This portion of the retina, which contains the neurovascular complex, is also fundamental to ensure the neural activity of the entire visual pathway.

However, to our knowledge, this is the first study showing a point-by-point correlation between the retinal sensitivity impairment and DCP abnormalities in patients with diabetic retinopathy and preserved visual acuity. Our group has already shown that. in diabetic patients with no sign of diabetic retinopathy, there was a capillary impairment at the level of DCP. However, the vessel density decrease could be also related to a neuroglial loss resulting in dysfunction of the interaction between neurons, glial cells, and vascular components [21].

Neural activity significantly correlates with local blood flow [22, 23], and alterations of the neuroglial tissue in the inner retina, found in these patients, might cause a secondary decrease in capillary flow density [24].

Interestingly, recently, different vascular retinal diseases have been correlated with similar findings.

A study by Kanakis et al. has shown that, in patients with branch retinal vein occlusion and macular ischemia, the DCP plays an important role on the metabolic demands of outer retina and, subsequently, an ischemia at the level of DCP has relevant influence on the health status of the photoreceptors [25].

Also, in patients with sickle cell retinopathy, examination confirmed that vascular flow voids on OCTA were correlated with focal decrease of retinal sensitivity by means of microperimetry (MP1) examination [26]. Specifically, the authors found that the area of dense scotoma corresponded to impairment of both the SCP and DCP, whereas the area of relative scotoma corresponds to compromise only in the DCP [26].

Nevertheless, to corroborate our hypothesis, there is the unquestionable certainty that, in patients with acute macular neuroretinopathy, a visual acuity and retinal sensitivity decreasing or scotoma reflects the DCP vascular insult, while the outer retinal alterations shown on SD-OCT appear only with time [27, 28].

Precisely, AMN might result from a simultaneous hypoperfusion more proximally at the level of the ophthalmic artery that causes a reduced perfusion of both the DCP and choriocapillaris [29].

Conversely, paracentral acute middle maculopathy, which is likely due to ischemia of the deep retinal circulation without a choriocapillary involvement, leaves a focus of inner retinal thinning or depression after resolution [29].

Although the analysis of the choroid was beside the aim of the study, focal choriocapillaris ischemia and diabetic choroidopathy cannot be totally excluded in the presence of photoreceptors damage. With regard to diabetic choroidopathy, a recent study from Borrelli et al. assessed the association between photoreceptor damage and choriocapillaris hypoperfusion in NPDR. They demonstrated that eyes with NPDR have macular hypoperfusion and reduced ellipsoid zone “normalized” reflectivity [30].

Furthermore, in eyes treated with panretinal photocoagulation, some changes might occur on choroidal thickness and choroidal blood flow as well.

However, it has been demonstrated that the panretinal photocoagulation leads to changes in biological response on microperimetry, which are slowly reversible over time. First, recently Lorusso et al. found that OCTA parameters were not significantly affected by PRP neither in short (1-month) nor long-term (6-month) follow-up [31]. Finally, Fawzi et al. showed an overall increase in the flow metrics of all capillary layers in the macula following PRP, unrelated to macular edema or thickening [32].

In our cohort of patients with proliferative diabetic retinopathy, PRP was performed mostly at the time of the diagnosis of retinal proliferations (range 9–5 years before the enrollment in the study).

Limitations of the present study are the relatively small number of patients. Furthermore, the retinal sensitivity is known to decrease slightly with age. In addition, because both outer retinal disruption and deep capillary nonperfusion are present in the same region, we speculated that these two retinal and vascular findings are mutually correlated. However, we cannot exclude that loss of retinal sensitivity may be only due to the outer retinal disruption. Finally, only in one case of our cohort, macular edema or microaneurysm/exudates at the edge of the area considered in the analysis might influence our findings.

## 6. Conclusions

This study shows that microperimetry is important to show the retinal sensitivity impairment in diabetic patients with macular ischemia. Additionally, we found that the areas with the decreased values of retinal sensitivity tightly correspond to the areas of decreased DCP density on OCTA. This reinforces the hypothesis that the health status of DCP and not only the choroid is important to the oxygen requirements of the photoreceptors in patients with diabetic macular ischemia. Also, the anatomical and functional consequences of these findings might help to explore the efficacy of new therapy in cases with relatively preserved visual acuity in clinical practice.

## Figures and Tables

**Table 1:** Demographic and clinical findings of study population.

Case	Sex/age, years	Duration of DM	Study eye	BCVA	DR stage	Laser treatment	HbA1c
1	M/69	12 years	LE	20/20	PDR	PRP	10.2
2	M/55	16 years	LE	20/20	Severe NPDR	None	8.6
3	M/55	6 years	LE	20/25	Severe NPDR	None	7.8
4	M/68	15 years	RE	20/25	PDR	PRP	9.3
5	M/62	15 years	LE	20/25	Severe NPDR	None	6.7
6	M/62	12 years	RE	20/40	PDR	PRP	10.1
7	M/56	12 years	LE	20/20	Severe NPDR	None	7.6
8	M/48	3 years	RE	20/20	PDR	PRP	8.7
9	F/67	15 years	RE	20/25	PDR	PRP	9.4
10	M/59	10 years	RE, LE	20/40	PDR	PRP	9.7
11	F/64	9 years	RE	20/40	Severe NPDR	None	7.3
12	M/61	8 years	RE, LE	20/25	Severe NPDR	None	6.5

Abbreviations: F = female; M = male; DM = diabetes mellitus; HbA1c = hemoglobin A1c value; RE = right eye; LE = left eye; PDR = proliferative diabetic retinopathy; NPDR = nonproliferative diabetic retinopathy; BCVA = best-corrected visual acuity; PRP = panretinal photocoagulation.

**Table 2:** Anatomical and functional findings of study population.

Case	SCP	DCP	FAZ-SCP	FAZ-DCP	CRT	MRS	FRS	Centralis 1°	Contour of the innermost retinal layers
1	−	+	+	+	230	9.2	5	3.1	Abnormal
2	−	+	+	+	249	15.9	13	15.8	Normal
3	−	+	+	+	265	7.4	6	6.7	Normal
4	−	+	−	+	342	15.4	13	14.6	Normal
5	+	+	+	+	299	13.6	12	15.6	Normal
6	−	+	−	+	298	11.6	10	11.3	Abnormal
7	+	+	+	+	246	12.9	11	13.5	Abnormal
8	−	+	−	+	296	15.8	13	15.9	Normal
9	+	+	+	+	245	13.4	11	8.4	Abnormal
10a	−	+	−	+	278	12.3	12	12.7	Normal
10b	−	+	−	+	267	13.4	13	12.5	Normal
11	+	+	+	+	296	11.7	11	10	Normal
12a	+	+	+	+	268	13.2	13	13.1	Normal
12b	+	+	+	+	273	12.9	12	13.2	Normal

Abbreviations: + = impairment; − = not involved; SCP = superficial capillary plexus; DCP = deep capillary plexus; FAZ = foveal avascular zone; CRT = central retinal thickness (microns); MRS = mean retinal sensitivity (decibel); FRS = foveal retinal sensitivity.

## Data Availability

The data used to support the findings of this study are available from the corresponding author upon request.

## Additional Points

Dr. Varano reports personal fees from Allergan, Bayer, Novartis, and SIFI outside the submitted work. Dr. Parravano reports personal fees from Allergan, Novartis, and Bayer outside the submitted work.
